# *Ligilactobacillus Salivarius* improve body growth and anti-oxidation capacity of broiler chickens via regulation of the microbiota-gut-brain axis

**DOI:** 10.1186/s12866-023-03135-x

**Published:** 2023-12-09

**Authors:** Jiajun Yang, Jing Wang, Zongliang Liu, Jun Chen, Jiajing Jiang, Minmeng Zhao, Daoqing Gong

**Affiliations:** 1 Jiangsu Key Laboratory of Animal genetic Breeding and Molecular Design, College of Animal Science and Technology, Yangzhou University, Yangzhou, 225009 Jiangsu China; 2 School of Animal Husbandry and Veterinary Medicine, Jiangsu Vocational College of Agriculture and Forestry, Jurong, 212400 Jiangsu China; 3 Hefei Zhien Biotechnology Company Limited, National University Science Park, No.602 of Huangshan Road, Hefei, 230031, 230001 Anhui Province China; 4 College of Animal Science and Food Engineering, Jinling Institute of Technology, Nanjing, 210038 Jiangsu China

**Keywords:** Probiotics, Broiler, Growth, Antioxidation, Gut-brain axis

## Abstract

Certain strains of probiotic bacteria can secret functional substances namely digestive enzymes and functional peptides to regulate physiological conditions such as digestion and anti-oxidation, which are often incorporated in industrial broiler chick production. However, few studies have detailed the action mechanisms and effects of these bacteria on regulating growth and anti-oxidation levels in broiler chickens. *Ligilactobacillus salivarius* is a strain of probiotic bacteria used as dietary supplement. In the present study, *Ligilactobacillus salivarius* was evaluated for its secreted digestive enzymes in *vitro*. To detailed evaluate the action mechanisms and effects of gastrointestinal tract (GIT) microbiota on alleviating anti-oxidation levels of broiler chickens through the gut-brain axis. *Ligilactobacillus salivarius* was cultured and supplemented in the food of broilers to evaluate the probiotic effect on growth and anti-oxidation by modulation of gut microbial composition and its functional metabolites using metagenomic and metabolomic assays. Biochemical results showed that *Ligilactobacillus salivarius* secreted digestive enzymes: protease, lipase, and amylase. Broiler chickens with *Ligilactobacillus salivarius* supplemented for 42 days, showed increased body weights, a reduced oxidative status, decreased malondialdehyde levels, and improved activities rates of total superoxide dismutase, glutathione peroxidase IIand IV improved. The microbial composition of caecum was more abundant than those broiler without probiotics supplementation, owing 400 of total number (489) of bacterial operational taxonomic units (OTU). The genera of *Lactobacillus*, *Megamonas*, *Ruminoccoccaceae*, *Ruminococcus*, *Alistipes* and *Helicobacter* shared the dominant proportion of *Candidatus* _*Arthromitus* compared with the control chickens. These functional bacteria genera assisted in the transportation and digestion of amino acids, carbohydrates, and ions, synthesis of cellular membranes, and anti-oxidation. Uncultured_organism_g_ *Anaerosporobacter*, *Lactobacillus salivarius*, uncultured_bacterium_g_ *Ruminococcaceae*_*UCG-014*, uncultured_bacterium_g_ *Peptococcus* were strongly and positively correlated with body growth performance and anti-oxidation. A metabonomic assay suggested that the secreted of gamma-aminobutyric acid and monobactam was metabolized according to the Kyoto Encyclopedia of Genes and Genomes analysis. In conclusion, *Ligilactobacillus salivarius* optimized microbial composition of the caecum and secreted functional peptides through gut-brain axis to improve the body growth and antioxidation of broiler chicken.

## Introduction

In broiler chicken industrial production, several unfavourable environmental conditions, including high concentrations of harmful gases, stocking density, heat, cold, and transportation, represent adverse factors for body health; these factors induct a decrease in growth and immunity, a disturbance of the gastrointestinal bacterial composition, and increased secretion of cortisol [1–3]. Probiotics are defined as live microorganisms that [4], when administered in adequate amounts, provide a myriad of benefits to body health, such as modulating immunity, improving the gut’s bacteria composition, and reducing oxidative levels through the secretion of functional substances [5, 6]. Probiotics are considered a useful additive substitute for antibiotics in industrial broiler chicken production [7, 8]. Some strains of probiotic bacteria can secrete digestive enzymes to help nutritional absorption including γ- aminobutyric acid [9], endorphin [10], and monobactam [11], which regulate brain functions through the gut-brain axis. The hormone secreted such as cortisol is affected by the composition of the microbiota to modulate anti-oxidation, as regulated by the hypothalamic–pituitary–adrenal axis [12].

*Ligilactobacillus salivarius* (*L*. *salivarius*) is a probiotic strain widely used in the food and diet of humans and animals. We have previously demonstrated dietary supplementation with 10^6^ colony-forming units of probiotics per gram of diet (CFU/g) [13]. Dietary supplementation with *L*. *salivarius* can decrease the level of stress-correlated oxidation in animal production [6, 13]. In addition, the gastrointestinal bacterial composition can be optimized via supplementation with *L*. *salivarius* [14]. However, its beneficial effects in broiler chickens remain unclear. The functional substances related to the antioxidant defence of *L*. *salivarius* are unknown. Moreover, the connection between bacterial composition in the gastrointestinal tract (GIT) and the secretion of hormones to regulate oxidation in broiler chickens has rarely been studied.

Metabolomic analyses have been widely used to identify unknown microbial metabolites [15]. Functional substances have often been identified, annotated, and quantified. Metagenomic assays are often employed to evaluate the intestinal bacterial composition and its relationship with body growth and other factors [16]. In this study, the secretion of digestive enzymes by *L*. *salivarius* was examined using a plate assay. Broiler chickens were fed a *L*. *salivarius* supplementation to increase their body weight. Biochemical analyses were performed to measure hormone secretion and oxidative status. To further detail the action pathway of *L*. *salivarius*, the optimized composition of the microbiota in the caecum was verified using a metagenomic assay, and metabolomics were used to identify functional substances. The purpose of our study was to elucidate the potential action mechanism of the microbiota of the GIT with *L*. *salivarius* supplementation through the gut-brain axis, as this mechanism may provide a new avenue for exploring the effects and potential connections of probiotic bacteria in practical use.

## Materials and methods

### Bacterial strain and digestive enzyme assay

*L*. *salivarius* was isolated by our research group at the School of Animal Husbandry and Veterinary Medicine, Jiangsu Vocational College of Agriculture and Forestry; it was then stored at the China General Microbiological Culture Collection Center (CGMCC), Beijing, China, with strain number CGMCC17718. The bacteria were cultured and colonised in de Man, Rogosa, and Sharpe medium (MRS) after inoculation at 1% incubation and 37℃ for 16 h of cultivation. After cultivation, the fermented medium was filtered at 0.45 μm to remove the bacteria. The fermentation liquid of *L*. *salivarius* was collected to measure the secretion of digestive enzymes. Whole milk powder (2 g) was added to the MRS medium to prepare the plated medium. An Oxford diffusion assay was employed using the Oxford diffusion method [14]. The biochemical colorimetric method was employed to quantify the capacity of the digestive enzyme; the live bacteria after fermentation were calculated and diluted to 10^9^ colony forming units per millilitre (CFU/mL), then filtered; the residual liquid was investigated for protease, lipase, and amylase activities secreted by *L*. *salivarius*. Detection kits were purchased from Nanjing Jiancheng Bioengineering Research Institute, Nanjing, China.

### Chickens, diet, and study design

To evaluate the supplemented efficiency of *L. salivarius*, 360 one-day-old Cobb broilers (average body weight 40.13 g purchased from Jiangsu Lihua Animal Husbandry Co., Ltd.) were randomly allocated to four groups with five replicates of 18 each. These were the control (CON), *L. salivarius* (LSA), vitamin C (VIT), and flavomycin-supplemented (FLA) groups. All animal management and euthanized in experiments were approved by the Institutional Animal Care and Use Committee of China and the Institution of Animal Science and Welfare of Jiangsu Province (no. IASWJSP202111739). The study was conducted in accordance to relevant guidelines and regulations. All efforts were obeyed the rules of animal welfare and were to minimize animal sufferings. All the authors confirm that the study is reported in accordance with ARRIVE guidelines (https://arriveguidelines.org).

Chickens in the control group were fed a basal diet, whereas those in the three treatment groups were fed a basal diet supplemented with *L. salivarius*, vitamin C [17] and flavomycin. The experimental diets were fed during two periods, designated as starter (days 0–21) and finisher (days 22–42). The basal diet composition, which did not include any probiotics or antibiotics, as well as the nutrient analysis results are shown in Table 1. All nutrients met or exceeded the nutrient requirements of the National Research Council (NRC, 2012) [18]. For chickens in the vitamin C group, 1.12 g of vitamin C (analytically pure) was diluted in 100 mL distilled water and blended with 3 kg of the basal diet. Thereafter, the mixed basal diet was added to a blender containing 97 kg of the basal diet. The blender was used for 10 min to ensure uniform mixing. For the *L. salivarius* group, 50 mL of *L. salivarius* fermentation liquid was measured separately and blended with 3 kg of the diet, followed by a mass diet of 97 kg. After preparation, 5 g of the diet was harvested to detect live bacteria using the plate method. The sample was treated with a series of ten dilutions; a 100 µL dilution was spread on the plate of the MRS solid medium. The number of live *L. salivarius* reached 2.5 × 10^6^ colony forming units per gram (CFU/g) of diet. The diet for the flavomycin group was prepared using 2 g of premixed food containing 20% flavomycin blended with 100 kg of the basal diet to reach a concentration of 4 mg/kg. All chickens were allowed ad libitum access to water and food throughout the experimental period.

**Table 1:** Nutrient analysis of the basic diet for broiler chicks

	Ingredient	Starter (0~21) %	Finisher (21~42) %
Item	Corn	58.12	61.75
	Soybean meal	29.15	26.45
	Fish powder	5.00	3.51
	Soybean oil	2.00	3.00
	Premix	5.00 ^a^	5.00 ^a^
	Dicalcium phosphorus	0.47	0.29
	Limestone	0.26	0
Calculated nutrient	Metabolizable energy (MJ /kg)	12.02	12.49
	CP	21	17.5
	Calcium	1	0.85
	Total phosphorus	0.68	0.65
	Available phosphorus	0. 5	0.42
	Lys	1.2	1.0
	Met	0.46	0.32

Note: The premix provides,

^a^Vitamins and trace elements per kg diet: Vitamin A (retinyl acetate) 9, 875 IU, Vitamin D_3_ (cholecalciferol) 3, 000 IU, Vitamin E (DL-ɑ-tocopheryl acetate) 20 IU, menadione 3.25 mg, Vitamin B_12_ (cyanocobalamin) 0.025 mg, thiamin 1.5 mg, riboflavin 5.0 mg, biotin 0.032 mg, folacin 1.25 mg, niacin 12 mg, pantothenic acid 12 mg, and pyridoxine 3.75 mg, manganese 100 mg, zinc 80 mg, iron 80 mg, copper 8 mg, iodine 0.15 mg, and selenium 0.15 mg

### Sample collection

The chicks in each replicate of each treatment group were weighed on day 42. Their daily dietary consumption was accurately recorded. After 42 d, three chickens with an average body.

weight in each replicate were selected (*n* = 5 × 3) and subjected to fasting for 12 h. Samples were harvested under general halothane anaesthesia (purchased from Pfizer Incorporated, USA) followed the dose of 0.65 mg per kilogram bodily wight. A 5 mL volume of blood was obtained from the wing vein and homogenised with heparin sodium to harvest plasma after centrifugation at 3000 rpm. The caecum tissues were removed under aseptic conditions, stored in sterile plastic tubes on ice, and immediately transported to our laboratory for deoxyribonucleic acid extraction. A segment of 3 cm ileum samples from the distal end of the ileum to the ileocecal orifice was collected and stored in 4℃, then the intestine was sectioned longitudinally and washed with cold physiological saline solution (4℃) to remove chyme. A 0.2 g sample of mucous membrane was shaved and ground to prepare a mucosal tissue homogenate.

### Hormonal levels and antioxidant status

Plasma samples were used to measure the hormone levels. Plasma cortisol [19], endotoxin [20], and antioxidative indices, namely malondialdehyde (MDA), superoxide dismutase (SOD), and activities of type II glutathione peroxidase (GPX) [21], were detected using enzyme-linked immunosorbent assay (ELISA). The activity rate of type IV GPX [22] in the caecal mucous membrane was measured using ELISA kits. These kits were purchased from Nanjing Jiancheng Bioengineering Institute (Nanjing, China).

### 16 S rDNA sequencing and analyses

Samples (0.20 g) of caecal chyme were collected and microbial DNA was extracted using a DNA isolation kit (TIANGEN Company, Beijing, China). The final DNA concentration and purity were determined using a NanoDrop 2000 UV-vis spectrophotometre (Thermo Scientific, Waltham, MA, USA), and DNA quality was determined using 1% agarose gel electrophoresis. V3-V4 hypervariable regions of the bacterial 16S rRNA gene were amplified with primers 338F (5’-ACTCCTACGGGAGGCAGCAG-3’) and 806R (5’-GGACTACHVGGGTWTCTAAT-3’) using a thermocycler PCR system (GeneAmp 9700, Applied biosystems, Foster City, CA, USA). PCR was prepared using the following procedures: 3 min of denaturation at 95 ℃; then, 27 cycles: 30 s at 95 ℃, 30 s of annealing at 55 ℃, 45 s of elongation at 72 ℃, and a final extension at 72 ℃ for 10 min. PCR was performed in triplicate in 20 µL mixtures containing 4 µL of 5×FastPfu Buffer, 2 µL of 2.5 mM dNTPs, 0.8 µL of each primer (5µM), 0.4 µL of FastPfu polymerase, and 10 ng of template DNA. The PCR products were extracted from a 2% agarose gel and further purified using the AxyPrep DNA Gel Extraction Kit (Axygen Biosciences, Union City, CA, USA) and quantified using a QuantiFluor™-ST (Promega, Madison, WI, USA) according to the manufacturer’s protocol. Purified amplicons were pooled at equimolar concentrations and paired-end sequencing was performed (2 × 300) on an Illumina MiSeq platform (Illumina, San Diego, CA, USA) according to standard protocols. Eight replicates were prepared for each group. Raw Illumina sequencing data were deposited in the Sequence Read Archive database (SRA) No. SRR18404173, of BioProject PRJNA817670.

Diversity metrics were calculated using the core diversity plugin in QIIME2 [23]. Feature-level alpha diversity indices and operational taxonomic units (OTUs) were used to estimate microbial diversity within an individual sample. A co-occurrence analysis between the anti-oxidative status, stress level and bacterial species in caecal chyme was performed by calculating Spearman’s rank correlations and producing network plots. Additionally, the potential Kyoto Encyclopedia of Genes and Genomes (KEGG) [24] orthologue functional profiles of the microbial communities were predicted using PICRUSt.

### Metabolomic assays of functional peptides

A fermented liquid without *L*. *salivarius* cells was prepared. Then, 100 µL of a fermented medium with *L*. *salivarius* were added to 800 µL of extract liquid composed of methanol and acetonitrile (volume ratio of 1:1) containing 0.02 mg/mL 2-chlorophenylalanine as an interior label. These substances were subsequently vortex mixed for 30 s and subjected to low temperature ultrasonic extraction (5℃, 40 kHz). The samples were refrigerated at -20℃ for 30 min, then centrifuged at 13,000 rpm/min for 30 min. Subsequently, the supernatant was reconstituted with a 120 µL water resolution containing 50% acetonitrile and transferred into a vial for ultra performance liquid chromatography tandem mass spectrometry (UPLC-MS) analysis [25]. Twenty microlitres of each sample to be tested were mixed with a quality control (QC) sample for error correction. Certain metabolising substances, such as monobactam, were identified by searching freely accessible KEGG, which is a database that integrates genomic, chemical, and systemic function information [26].

To qualify the concentrations of gamma-aminobutyric acid (ɤ-GABA) and monobactam in the fermented liquid of *L*. *salivarius*, the standard of ɤ-GABA (Alladdin Company, USA) and monobactam (Pfizer, USA) were purchased and diluted into 1ng/mL, 5ng/mL, 10ng/mL, 50ng/mL, 100ng/mL, and 1000ng/mL concentrations to establish the standard curve. The assays were performed following the UPLC-MS procedure [27].

### Statistical analyses

Body weight and DNA sequencing data (Shannon index) were subjected to one-way ANOVA using the GLM procedure in SPSS, with significance reported at *P* < 0.05. The means were further separated using Duncan’s multiple range test [28]. A *P*-value of less than 0.05 was considered statistically significant.

## Results

### Secretion of digestive enzymes

**Fig. 1:**
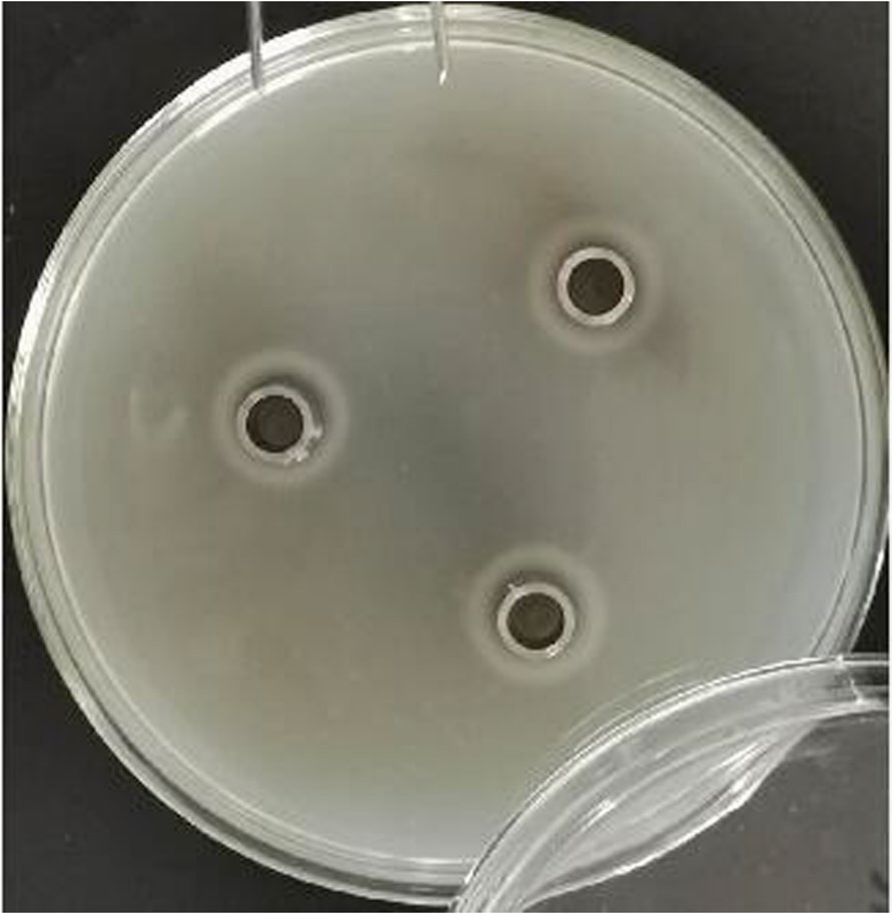
The Oxford diffusion assay on the digestive enzymes secreted by *Ligilactobacillus salivarius.* The obvious dissolved zone can be observed around the Oxford cap. The diameter of the zones was 18.29 mm

An obvious zone of plaque was observed on the milk MRS medium plate, as indicated by the Oxford diffusion results shown in Fig. 1. An obvious dissolved zone was observed in the outer space of the Oxford cap. The diameters of the zones reached 18.29 mm. The capacities of protease, lipase, and amylase secreted by *L*. *salivarius* were 178.89U/mL, 698.29U/mL, and 532.43U/mL, respectively at 10^9^ CFU/mL.

### Impact on growth performance

**Fig. 2:**
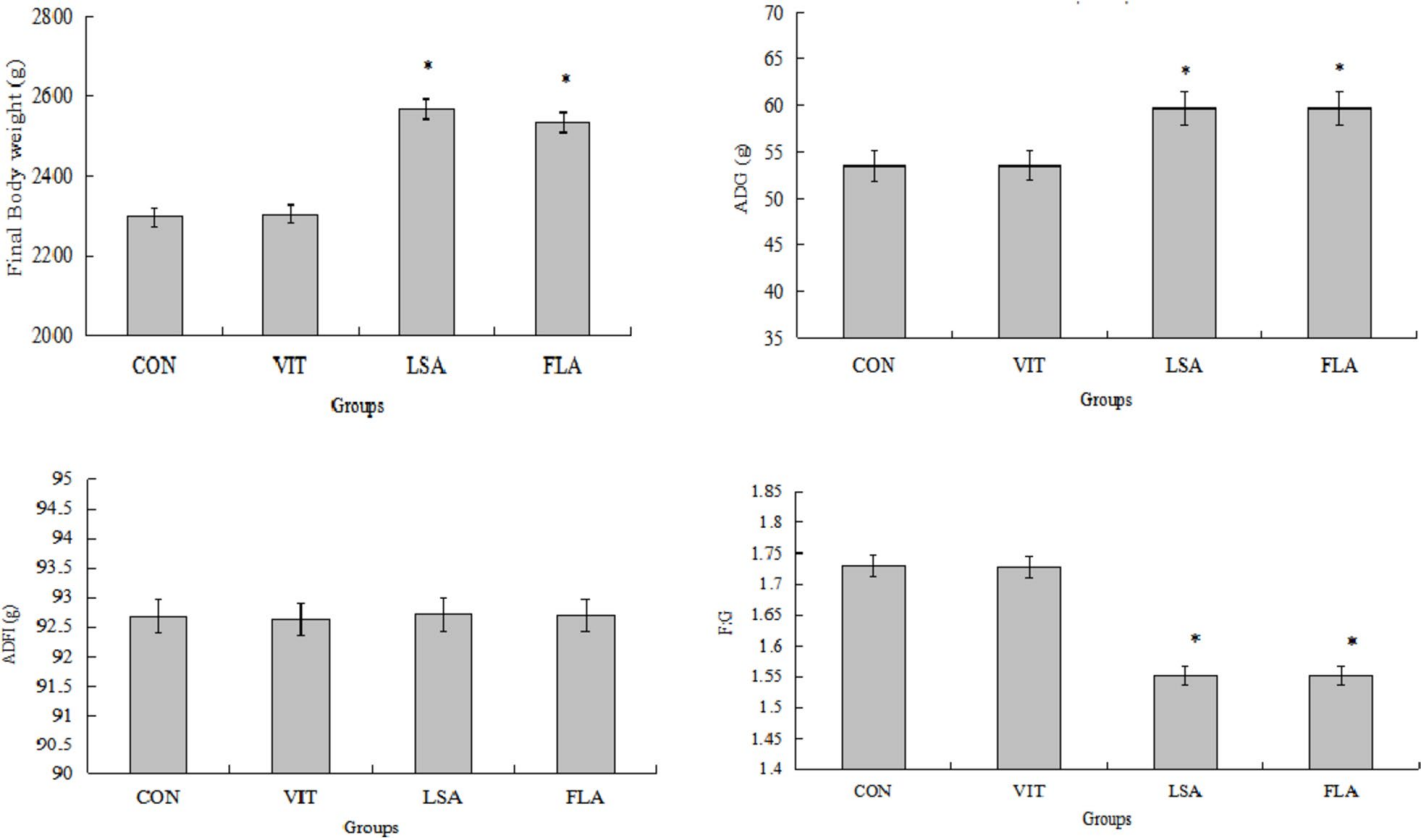
Effects of different treatments on growth performance of broiler chicken. The final body weight, average daily gain (ADG), average daily feed intake (ADFI), and ratio of feed: gain (F: G). Chicks were treated with control (CON), vitamin C (VIT), *Ligilactobacillus* *salivarius* (LSA), and flavomycin (FLA) after 42 days. Data was statistically processed as one-way ANOVA using the GLM procedure of SPSS, with significance reported at *P* < 0.05. Bars represent mean ±S.E. * is significantly different from those without (*P*<0.05). Figure 2**a**. Effects of different treatments on chicken body weight. Figure 2**b**. Effects of different treatments on chicken average daily gain (ADG). Figure 2**c**. Effects of different treatments on chicken average daily feed intake (ADFI). Figure 2**d**. Effects of different treatments on chicken ratio of feed: gain (F: G)

The growth performance is shown in Fig. 2. After supplementation with *L*. *salivarius* for 42 d, the broiler chickens displayed a higher average body weight than those of the control by 268.93 g (Fig. 2a) (*P* < 0.05). However, this was not significantly different from the average weight of chickens who received the flavomycin supplementation (*P* > 0.05). Body weights in the vitamin C supplanted groups were not significantly different from that of the control group (*P* > 0.05). The average daily gain was the same (Fig. 2b). Broiler chickens inoculated with *L*. *salivarius* and flavomycin were higher than those in the control (*P* < 0.05). There were no differences in the average daily feed intake among the treatments (Fig. 2c) (*P* > 0.05). The ratio of feed:gain in the vitamin C and control groups was nearly 0.18 units higher than those in chicks with the *L*. *salivarius* and flavomycin supplements (Fig. 2d) (*P* < 0.05).

### Hormonal levels and antioxidant status

Cortisol levels in the plasma of chicks are listed in Table 2, with *L*. *salivarius* supplementation significantly decreasing in all treatments (*P* < 0.01). The result in the vitamin C group was also lower than those in the control and flavomycin groups (*P* < 0.01). The levels of endotoxins were also reduced in the *L*. *salivarius*-supplemented group (*P* < 0.01).

**Table 2:** Effects of different treatment on body anti-oxidation and hormonal levels

Groups	Malondialdehyde Μmol/L	Superoxide dismutase U/mL	Glutathione peroxidase II U/mL	Glutathione peroxidase U/mL	Cortisol ng /mL	Endotoxin EU/mL
C1	8.15^a^	89.55^a^	518.75^ab^	104.62^a^	152.65^A^	0.42 ^A^
C2	6.83^b^	112.42^b^	499.50^a^	116.51^b^	141.87^B^	0.34 ^B^
C3	5.07^c^	123.79^c^	533.75^bc^	136.55^b^	123.59^C^	0.29 ^C^
C4	6.86^b^	109.89^b^	549.00^c^	105.87^a^	149.89^A^	0.43 ^A^
s.e.m.	0.59	4.89	24.23	9.27	4.67	0.02
P	0.27	0.37	0.19	0.33	0.06	0.05

Mean value was significantly different from that of the control group by one-way ANOVA followed by Tukey’s multiple comparison tests: the different superscript capital letters in the same color of column mean significant difference at 0.01 levels (*P* < 0.01), lowercase letters means.significant difference at 0.05 levels (*P* < 0.05)

Plasma antioxidant indices were also measured. The MDA content in chickens supplemented with *L*. *salivarius* significantly decreased, and the activity rates of SOD and type II GPX were higher than those of the vitamin C and flavomycin groups (*P* < 0.05), which were enhanced compared with the control (*P* < 0.05). The activity rates of type IV GPX in the *L*. *salivarius*-and vitamin C-supplemented groups also improved compared to those of the control and flavomycin groups (*P* < 0.05).

### Optimized bacterial composition in cecum

**Fig. 3:**
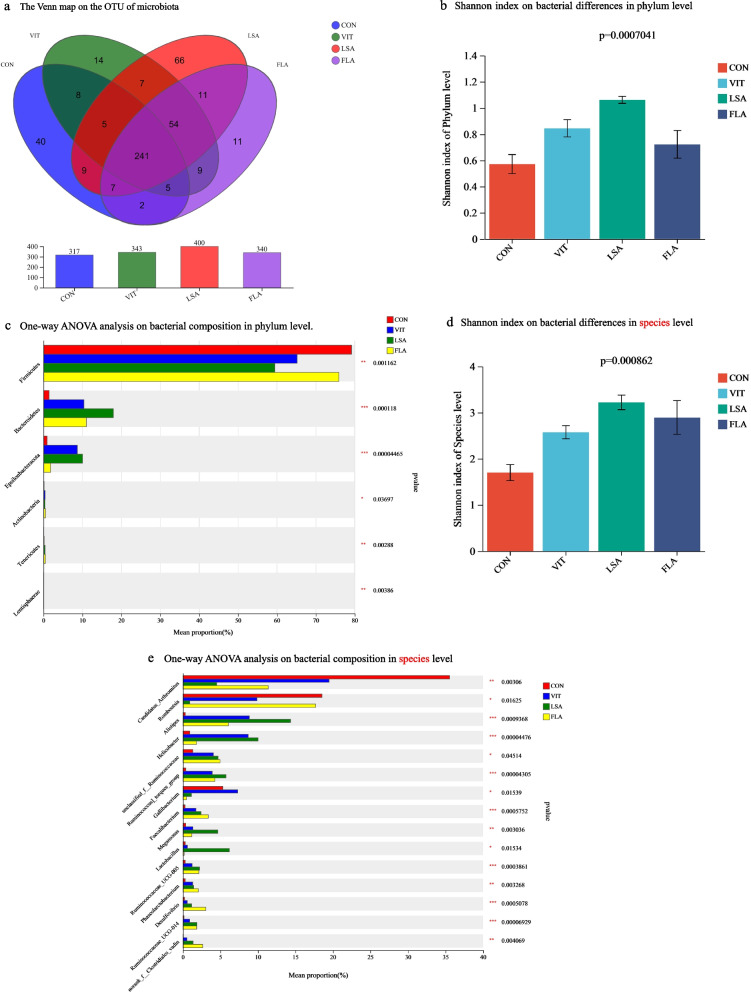
The overall profile of microbiota composition in cecal digesta of chicks. Figure 3**a** The Venn map on the OTU of microbiota in digesta of cecum. Figure 3**b** Shannon index on bacterial differences in phylum level. Figure 3**c** One-way ANOVA analysis on bacterial composition in phylum level. Figure 3**d** Shannon index on bacterial differences in species level. Figure 3**e** One-way ANOVA analysis on bacterial composition in species level.* in same column means *P* < 0.05, ** means *P* < 0.01, *** means *P* < 0.001

 The caecal chyme microbial 16 S rRNA metagenome was sequenced, and the results are shown in Fig. 3. The bacterial composition in the *L*. *salivarius* group was the highest, and the number of OTUs reached 400, covering 81.79% of the total of 489 (Fig. 3a). The number of patients in the control group was 317, which was the lowest of all groups (*P* < 0.001). The OTU diversity was in the order *L*. *salivarius* > vitamin C > flavomycin > control. The composition of bacteria in the caecum showed significant differences between *L*. *salivarius* and flavomycin supplementation through alpha diversity (Shannon index) at both the phylum and genus levels (Fig. 3b and d). At the phylum level, the dominant bacteria in the control group were *Firmicutes*, and there were a few other bacterial phyla observed. The chicks in the supplemented groups had higher abundances of *Epsilonbacteraeota* (*P* < 0.001), *Actinobactria* (*P* < 0.05), *Tenericutes* (*P* < 0.01), and *Lentisphaerae* (*P* < 0.01), compared to the controls. The bacterial abundance in the *L*. *salivarius* group was the highest. No significant differences were observed between the vitamin C and flavomycin groups. At the species level, the abundance of the three supplementary groups improved, and the Shannon indices shown in Fig. 3c suggested an increased diversity (*P* < 0.01). The richness of uncultured_bacterium_g*Faecalibacterium*, uncultured _bacterium_g_*Megamonas*, gut_*metagenome*_g_*Desulfovibrio*, *Ruminococcaceae*, and unclassified_f_*Lachnospiraceae* were higher in the three supplementary groups than in the control groups. The abundances of unclassified_f_*Lachnospiraceae* and uncultured _bacterium_g_*Megamonas* were the highest (*P* < 0.01). gut*metagenome*, uncultured_bacterium_g_*Megamonas Ruminococcaceae* in the *L*. *salivarius* groups (*P* < 0.01). Uncultured bacterium_g_*Romboutsia* and *Desulfovibrio* contained in flavomycin group were higher than those in the other groups (*P* < 0.01). The abundance of Gallibacterium_anatis was the highest in the vitamin C group.

### Functions of the optimised bacterial composition

**Fig. 4:**
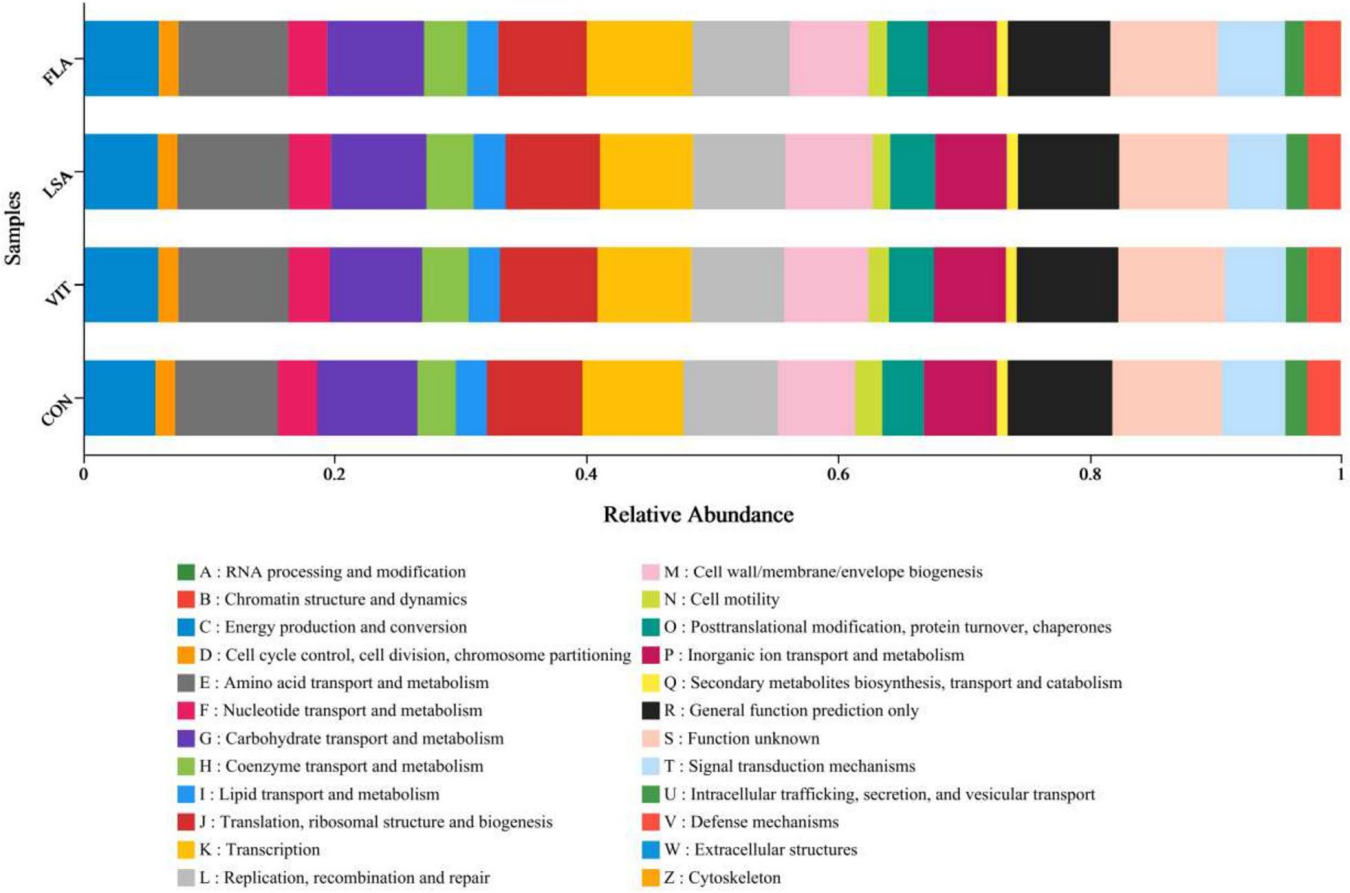
The functional analysis on bacterial composition. Kyoto Encyclopedia of Genes and Genomes (KEGG) pathway function classification. KEGG is a database resource that integrates genomic, chemical, and systemic functional information. STAMP software was applied to detect the differentially abundant KEGG pathways among groups with false discovery rate correction. Certain functions related genes: energy production and conversion, nucleotide transport and metabolism, cell membrane bio-genesis were increased

 The functions of the optimised bacterial genes were predicted and classified using the KEGG pathway, as shown in Fig. 4. Certain function-related genes, such as energy production and conversion, nucleotide transport and metabolism, and cell membrane biogenesis were increased in *L*. *salivarius* and vitamin C-supplemented groups compared with chicks in the control group. The abundances of two kinds of genes, transcription and signal transduction mechanisms, were the highest in the *L*. *salivarius* group compared to the other three groups.

**Fig. 5:**
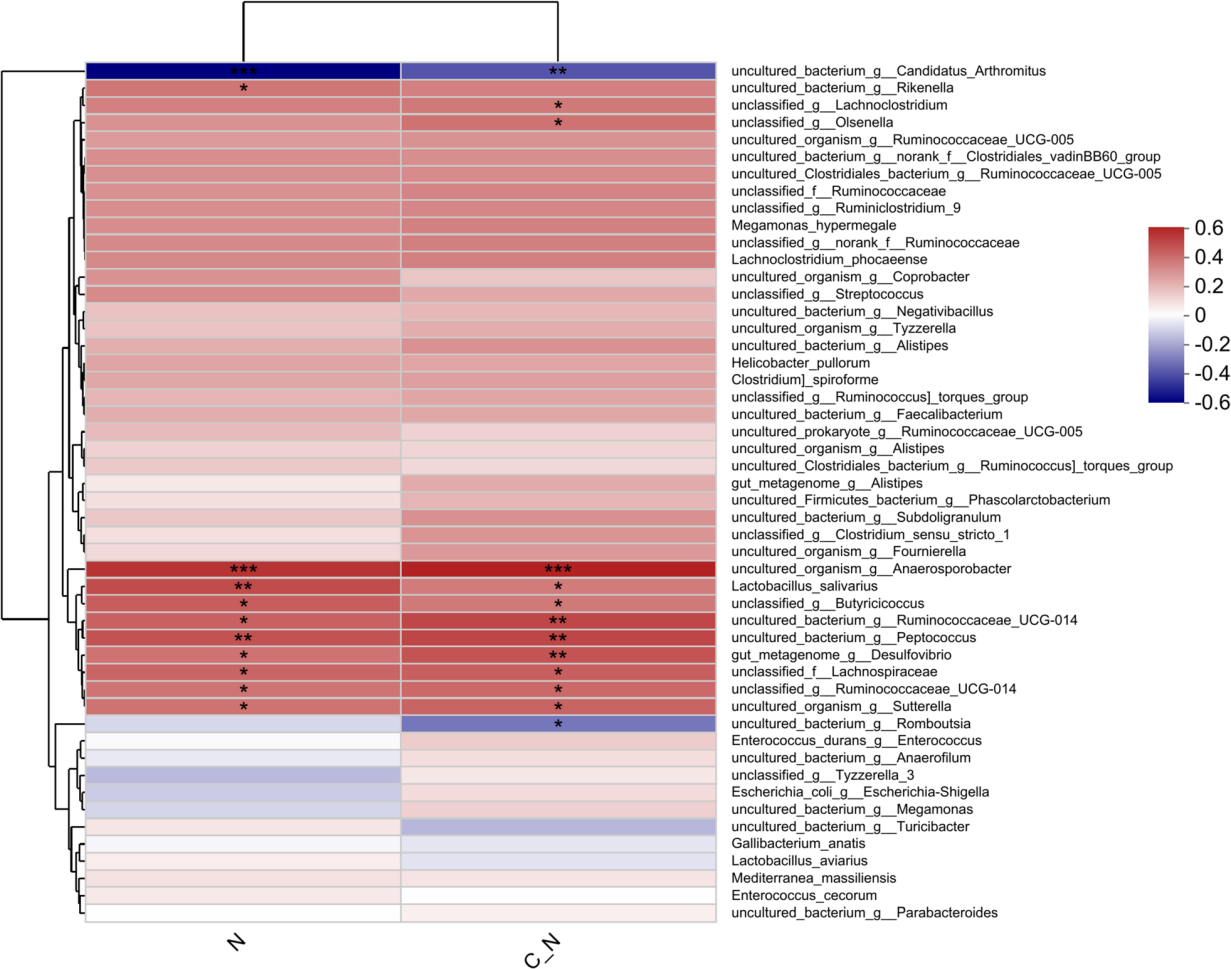
Spearman’s correlation analysis for level of cortisol, anti-oxidation with species of bacteria in cecum (N and C_N represented the level of cortisol and anti-oxidation). * means *P*<0.05, ** means *P*<0.01, *** means *P* < 0.001. Relative abundance is indicated by a color gradient from green to red, with green representing low abundance and red representing high abundance. Levels of body stress and anti-oxidation was negative corrected with uncultured organism_g_*Anaerosporobacter*, uncultured bacterium_g_*Ruminococcaceae*_UCG-014, unculture_bacterium_g_*Peptococcus*, gut_*metagenome*_g_*Desulfovibrio*, *Lactobacillus salivarius*, unclassified _g_*Butyricicoccus*, unclassified_f_*Lachnospiraceae*, unclassified_g_*Ruminococcaceae*_UCG-014, uncultured organism_g_*Sutterella*

 To discern the correlation between the species of functional bacteria, body growth, and antioxidation, Spearman’s analysis was conducted, as shown in Fig. 5. The levels of body growth and anti-oxidation were negatively corrected with uncultured organism_g_*Anaerosporobacter* (*P* < 0.001), uncultured bacterium_g_*Ruminococcaceae*_ UCG-014, uncultured_bacterium_g_*Peptococcus*, gut_*metagenome*_g_*Desulfovibrio* (*P* < 0.01), *Lactobacillus salivarius*, unclassified_g_*Butyricicoccus*, unclassified_f_*Lachnospiraceae*, unclassified_g_*Ruminococcaceae*_UCG-014, and uncultured organism_g_*Sutterella* (*P* < 0.05).

### Functional metabolites

**Fig. 6:**
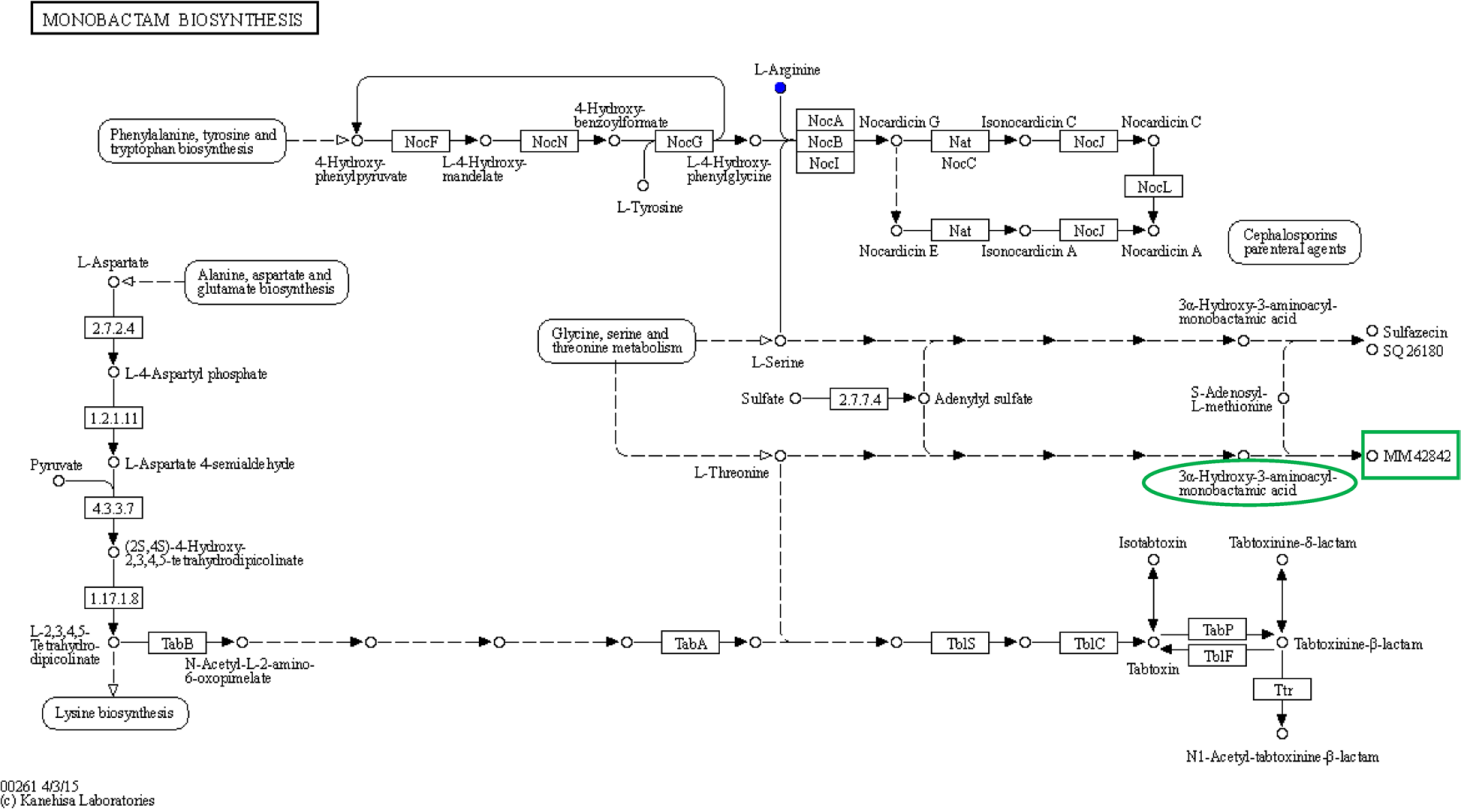
The profile of Monobactam on *Ligilactobacillus salivarius*. The biosynthesis pathway was annotated depending on KEGG. The graph of metabolic pathway was found through enriched of KEGG metabolic pathway after collecting the metabolites through UPLC-MS detection. Monobactam produced by *L. salivarius* and their pathway were manifested comprehensively. The pathway was originated from the biosynthesis of phenylalanine, tyrosine, and tryptophan through the cascade catalyzed reactions, which divided into two pathways, one is the metabolism of L-Arginine, the other way was continuously biosynthesized and merged into metabolism of glycine, serine, and threonine. One pathway of alanine, aspartate, and glutamate biosynthesis is also involved to synthesize 3α-Hydroxy-3-aminoacyl-momobactamic acid the precursor of monobactam, and finish the synthesis in further step

 Functional metabolites secreted by *Ligilactobacillus salivarius* were detected using UPLC-MS; ɤ-GABA and monobactam were measured. The effective concentrations of ɤ-GABA and monobactam reached 382.19 and 456.93 ug/L, respectively. The biosynthetic pathway was characterised using the KEGG classification, as shown in Fig. 6. The pathway originate from the biosynthesis of phenylalanine, tyrosine, and tryptophan through cascade-catalysed reactions. These reactions are divided into two pathways: the metabolism of L-arginine, and continuous syntheses that were merged into the metabolisms of glycine, serine, and threonine. One pathway of alanine, aspartate, and glutamate biosynthesis also influenced the synthesis of 3α-Hydroxy-3-aminoacyl-momobactamic acid, the precursor of monobactam. The product was completed in a further step.

## Discussion

Numerous studies have suggested that probiotic bacteria can promote livestock animals production. However, some studies have reported contrasting results [29, 30]. Therefore, the benefits of candidate probiotic bacteria as a supplement for chicks should be further evaluated. *L*. *salivarius* is a probiotic bacterium that is often used in the diets of humans and animals [31, 32]. *L*. *salivarius* was isolated and supplemented in the diet to detect effects on performance and the action pathway to evaluate its use in industrial broiler chicken production.

The digestive enzymes metabolised by *L*. *salivarius* were first measured. These measurements suggested that three types of enzymes–protease, lipase, and amylase–could be secreted. In broiler chickens, insufficient feed digestion and nutrition absorption cause a waste of feed some nutrients in the diet cannot be digested or excreted in faeces and are, therefore, wasted [33]. When supplemented with a dose of 10^6^ CFU/g *L*. *salivarius* in the diet, digestive enzymes can be secreted by gut bacteria and used to promote digestion in broiler chicks, thereby improving digestion efficiency and nutrition utilization [6, 14].

The digestive enzymes produced represent one of several advantages of *L*. *salivarius* [31]. The optimal bacterial composition in the GIT was determined. Results of alpha diversity on bacterial community showed that the OTUs from chicks supplemented with *L*. *salivarius* were richest, both in phylum and species levels. Increased abundance and types of bacteria at both the phylum and species levels were detected in caecal chyme with the *L*. *salivarius* supplementation. The bacterial proportions were evenly distributed. The proportion of *Firmicutes* to *Bacteroidetes* was reduced to 3.5:1, which was healthier than the control and appropriate for intestinal digestion and metabolism [32]. The diversity of bacterial differences at the species level is also shown. Unclassified_f_*Lachnospiraceae* and uncultured _bacterium_g_*Megamonas* were the most abundant in the *L*. *salivarius* group. *Faecalibacterium*, *Megamonas*, gut_*metagenome*_g_*Desulfovibrio*, *Ruminococcaceae*, and unclassified_f_*Lachnospiraceae* increased in richness. All these improved bacterial species demonstrate improved digestion and absorption [34, 35], leading to enhanced growth performance and immunity [36, 37].

The correlation analysis of bacterial species suggested that the increased abundances of *Ruminococcaceae*_UCG-014, unculture_bacterium_g_*Peptococcus*, gut_*metagenome*_g_ *Desulfovibrio*, unclassified_g_*Butyricicoccus*, and unclassified_f_*Lachnospiraceae* led to improved body growth and lower anti-oxidation levels. The improved bacterial composition at both the phylum and species levels was crucial for the probiotic role of *L*. *salivarius* [31].

The prediction for genetic functions of the composition of the microbiota indicated that with *L*. *salivarius* supplementation, energy production and conversion, nucleotide transport and metabolism, cell membrane biogenesis, transcription, and signal transduction mechanisms improved. Dietary supplementation with *L*. *salivarius* can regulate body stress and minimize oxidative damage to cellular membranes. The bacteria were previously recognised by toll like receptors (TLRs) of the microbiological pattern recognition receptor, activated nuclear transcription factor kappa B (NF-κB), through the classic signalling pathway TLR-NF-κB [38]. The gut-brain axis was regulated by the microbial composition of the GIT. Oral supplementation of *L*. *salivarius* regulated brain functions.

Body hormone secretion was regulated by brain. In suitable physiological condition, levels of cortisol and adrenaline synthesized in adrenal gland will be up-regulated to overcome the stress [39, 40], accompanied with high levels of bodily anti oxidative status. In industrial breeding and production, unfavourable environmental conditions often cause adverse factors to chicks [41]. Supplemented with *L*. *salivarius* can reduce the secretion of cortisol and oxidative levels. To unveil the related metabolites produced by the bacteria, the metabolomic assays were employed, and the metabolic profiles of *L*. *salivarius* in relation to stress mitigation were also studied. The secretions of ɤ-GABA and monobactam appear to represent another mitigation factor. ɤ-GABA is a neurotransmitter in the central nervous system [42], and performs the biological roles of improving sleep quality, inhibiting excitement, and lowering blood pressure; its action mechanisms have been widely clarified, leading to its widespread usage [43]. Monobactam is another functional peptide secreted by *L*. *salivarius* that facilitates antihypertension, analgesia, and anti-convulsion benefits [44]. It has been used as an antibiotic drug in recent clinical studies [45]. The production of monobactam via the chemical pathway is expensive and intensive, and can be synthesised through microbiological paves [46]. The results showed that the metabolisms of phenylalanine, tyrosine, tryptophan, L-arginine, glycine, serine, and threonine all influenced monobactam synthesis.

These two peptides are secreted into the gut during bacteria colonisation and are then assimilate into the blood and transported to the brain. This influences hormone secretion through the gut-brain axis [47]. Hence, the stress-related hormones cortisol and endotoxin decreased in chicks supplemented with *L*. *salivarius*. The results of the antioxidative indices SOD and types II, and IV of GPX indicated an improved anti-oxidation status [48]. The mitigated stress induced by higher body antioxidation is attributed to a lower oxidative status [49]. The concentration of MDA, an oxidation productin plasma also declined [50].

## Conclusion

**Fig. 7:**
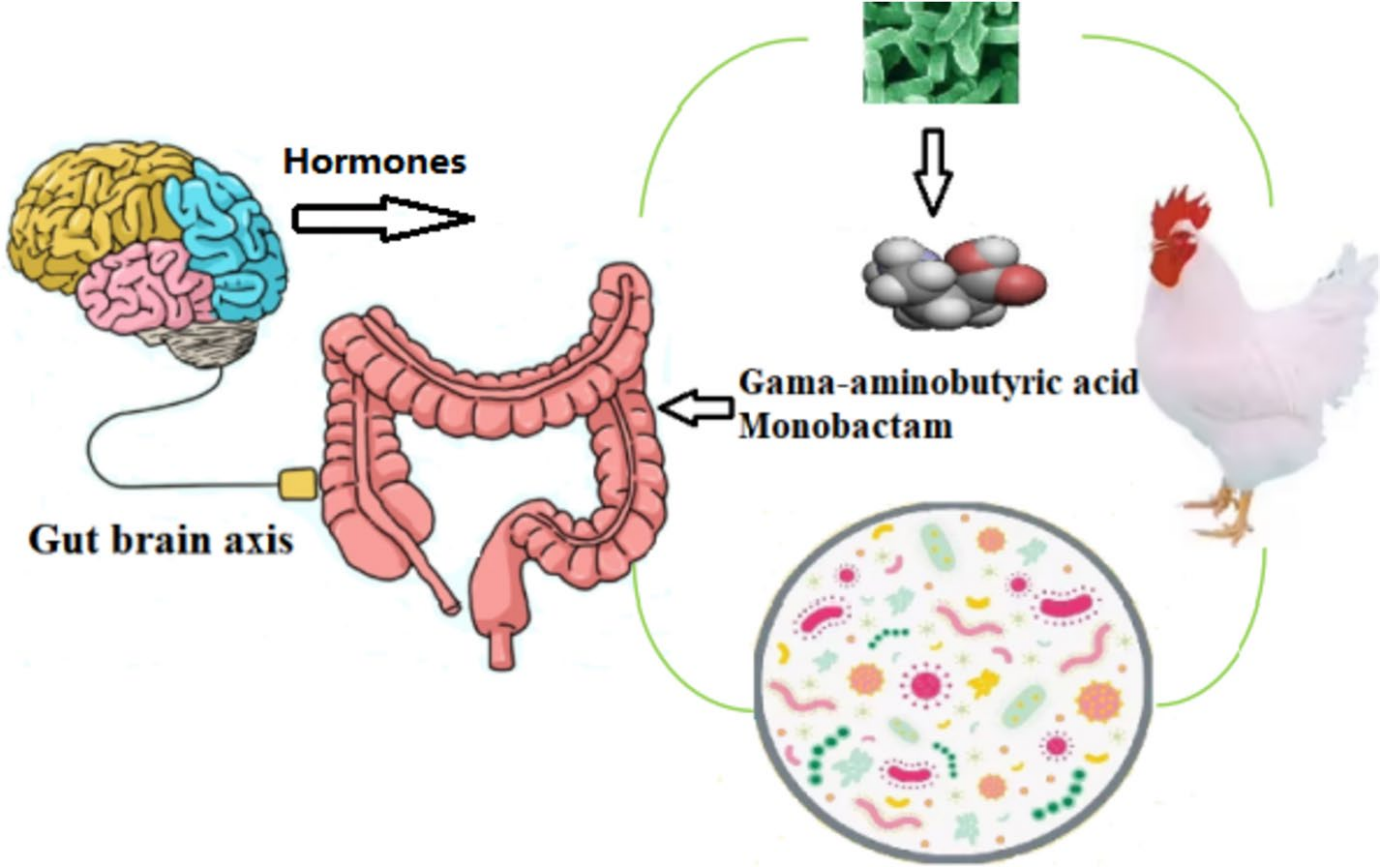
The graph of *Ligilactobacillus salivarius* The graph of *Ligilactobacillus salivarius* regulate stress of broiler chicken via gut brain axis

*Ligilactobacillus salivarius* secret digestive enzymes in its metabolism. A dietary supplementation with 2.5 × 10^6^ CFU/g *L*. *salivarius* can help broiler chicken digest and nutrition absorption, establish optimized caecal bacterial composition, also its syntheses of ɤ-GABA and monobactam absorbed into brain to modulate the secretion of stress-hormone cortisol though the gut-brain axis, reduce the oxidative levels, inducing to improved body growth (Fig. 7). Our study unveiled the effects and elucidated the potential action mechanism of *L*. *salivarius* supplementation through the gut-brain axis, which provided a new avenue for evaluating and exploring the potential action of probiotics in practical use.

## Data Availability

The Illumina sequencing raw data have been deposited into the Sequence Read Archive database (SRA) of National Center for Biotechnology Information (NCBI), deposited No. SRR18404173, BioProject PRJNA817670.
